# Putative endogenous filovirus VP35-like protein potentially functions as an IFN antagonist but not a polymerase cofactor

**DOI:** 10.1371/journal.pone.0186450

**Published:** 2017-10-17

**Authors:** Tatsunari Kondoh, Rashid Manzoor, Naganori Nao, Junki Maruyama, Wakako Furuyama, Hiroko Miyamoto, Asako Shigeno, Makoto Kuroda, Keita Matsuno, Daisuke Fujikura, Masahiro Kajihara, Reiko Yoshida, Manabu Igarashi, Ayato Takada

**Affiliations:** 1 Division of Global Epidemiology, Research Center for Zoonosis Control, Hokkaido University, Sapporo, Japan; 2 Laboratory of Microbiology, Department of Disease Control, Faculty of Veterinary Medicine, Hokkaido University, Sapporo, Japan; 3 Global Station for Zoonosis Control, Global Institution for Collaborative Research and Education, Hokkaido University, Sapporo, Japan; 4 Division of Infection and Immunity, Research Center for Zoonosis Control, Hokkaido University, Sapporo, Japan; 5 School of Veterinary Medicine, the University of Zambia, Lusaka, Zambia; Division of Clinical Research, UNITED STATES

## Abstract

It has been proposed that some non-retroviral RNA virus genes are integrated into vertebrate genomes. Endogenous filovirus-like elements (EFLs) have been discovered in some mammalian genomes. However, their potential roles in ebolavirus infection are unclear. A filovirus VP35-like element (mlEFL35) is found in the little brown bat (*Myotis lucifugus*) genome. Putative mlEFL35-derived protein (mlEFL35p) contains nearly full-length amino acid sequences corresponding to ebolavirus VP35. Ebola virus VP35 has been shown to bind double-stranded RNA, leading to inhibition of type I interferon (IFN) production, and is also known as a viral polymerase cofactor that is essential for viral RNA transcription/replication. In this study, we transiently expressed mlEFL35p in human kidney cells and investigated its biological functions. We first found that mlEFL35p was coimmunoprecipitated with itself and ebolavirus VP35s but not with the viral nucleoprotein. Then the biological functions of mlEFL35p were analyzed by comparing it to ebolavirus VP35s. We found that the expression of mlEFL35p significantly inhibited human IFN-β promoter activity as well as VP35s. By contrast, expression of mlEFL35p did not support viral RNA transcription/replication and indeed slightly decrease the reporter gene expression in a minigenome assay. These results suggest that mlEFL35p potentially acts as an IFN antagonist but not a polymerase cofactor.

## Introduction

Ebolaviruses are members of the family *Filoviridae* and cause severe hemorrhagic fever in humans and nonhuman primates. Five distinct species are known in the genus *Ebolavirus*: *Zaire ebolavirus*, *Sudan ebolavirus*, *Taï Forest ebolavirus*, *Bundibugyo ebolavirus*, and *Reston ebolavirus*, represented by Ebola virus (EBOV), Sudan virus (SUDV), Taï Forest virus (TAFV), Bundibugyo virus (BDBV), and Reston virus (RESTV), respectively. RESTV has never caused lethal infection in humans. Filoviruses infect a variety of cell types *in vitro* [1–6], and several cellular factors have been shown to be involved in filovirus replication in host cells [7, 8]. However, the details of the mechanisms underlying the cell tropism and pathogenicity of filoviruses have not been fully elucidated yet.

It has been reported that some non-retroviral RNA virus gene sequences are found in vertebrate genomes [9, 10]. Although the biological significance of these genomic sequences is largely unknown, it was particularly noted that expression of an endogenous bornavirus-like nucleoprotein element (EBLN) found in the ground squirrel genome, which is one of such host genomic sequences, conferred resistance of oligodendroglia cells to the virus infection [11]. Recent studies have further reported that transcription of human EBLN-1 is responsible for regulating gene expression of host cells [12–14]. These observations suggest that the expression of EBLNs has some beneficial roles like endogenous retroviruses in animal genomes (e.g., *syncytin-1* and *-2*, *syncytin-A*, *-B*) [15–17]. On the other hand, hyperexpression of an endogenous retrovirus, multiple sclerosis-associated retrovirus (MSRV) whose envelope gene shares >93% similarity with *syncytin-1*, is thought to be involved in multiple sclerosis [18, 19]. It has also been reported that the expression of syncytin-1 or the MSRV envelope protein in astrocytes or peripheral blood mononuclear cells is associated with a proinflammatory and autoimmune cascade [20, 21]. These observations suggest that endogenous retroviruses have a variety of effects on cell physiology.

Endogenous filovirus-like elements (EFLs) have also been discovered in several mammalian (e.g., tarsier, opossum, mouse, rat, and bat) genomes [10, 22–24], however, the potential roles of EFLs in the ebolavirus replication have not been elucidated. It is speculated that some of the EFLs (mlEFLN, mlEFL35, saEFLN, and meEFLN) have been present in the host genome for over 20 million years [10] and the presence of EFLs in host genomes may suggest a correlation with cellular susceptibility to ebolavirus infection [10, 22]. Of these EFLs, an endogenous filovirus VP35-like element found in the little brown bat (*Myotis lucifugus*), mlEFL35, has a nearly full-length open reading frame (ORF) corresponding to the VP35 gene [10]. EBOV VP35 has been shown to bind double-stranded RNA (dsRNA) and inhibit type I interferon (IFN) production [25]. IFNs are a group of signaling proteins released from host cells in response to the presence of several pathogens. Viral infections commonly induce production of type I IFNs, which interfere with viral replication in infected cells, resulting in the restriction of virus propagation. EBOV replication was also shown to be inhibited by the IFN response induced by the retinoic acid-inducible gene-I (RIG-I) activation in cell culture [26]. It has been shown that ebolavirus VP35s impair human IFN-β promoter activation by inhibiting the function of RIG-I, IFN-β promoter stimulator 1 (IPS-1), and TANK-binding kinase 1 (TBK1) [27]. Furthermore, EBOV possessing VP35 with reduced ability to bind dsRNA is significantly attenuated in mice [28]. Thus, VP35s have been thought to be an important factor in the pathogenesis of ebolavirus infection. In addition to its function as an IFN inhibitor, VP35 is known as an essential cofactor in the viral polymerase complex of ebolaviruses [29]. Four viral proteins: nucleoprotein (NP), VP35, VP30, and RNA-dependent RNA polymerase (L) are major structural components of the nucleocapsid complex and are involved in viral replication and transcription [29, 30]. VP35 interacts with NP and L in this complex and both VP35-NP and VP35-L interactions are believed to be essential for viral RNA synthesis [29, 31–34].

To investigate potential functions of mlEFL35, we constructed plasmids expressing putative mlEFL35-derived protein (mlEFL35p) in cultured cells and performed functional analyses to evaluate the potential of mlEFL35p as an IFN antagonist and/or polymerase cofactor. Here we show that mlEFL35p, as is the case with EBOV VP35, inhibits the RIG-I-mediated signaling pathway and the production of IFN-β but does not act as a polymerase cofactor or dominant negative inhibitor.

## Results

### mlEFL35p and ebolavirus VP35s partially share the primary structure

**Fig 1:**
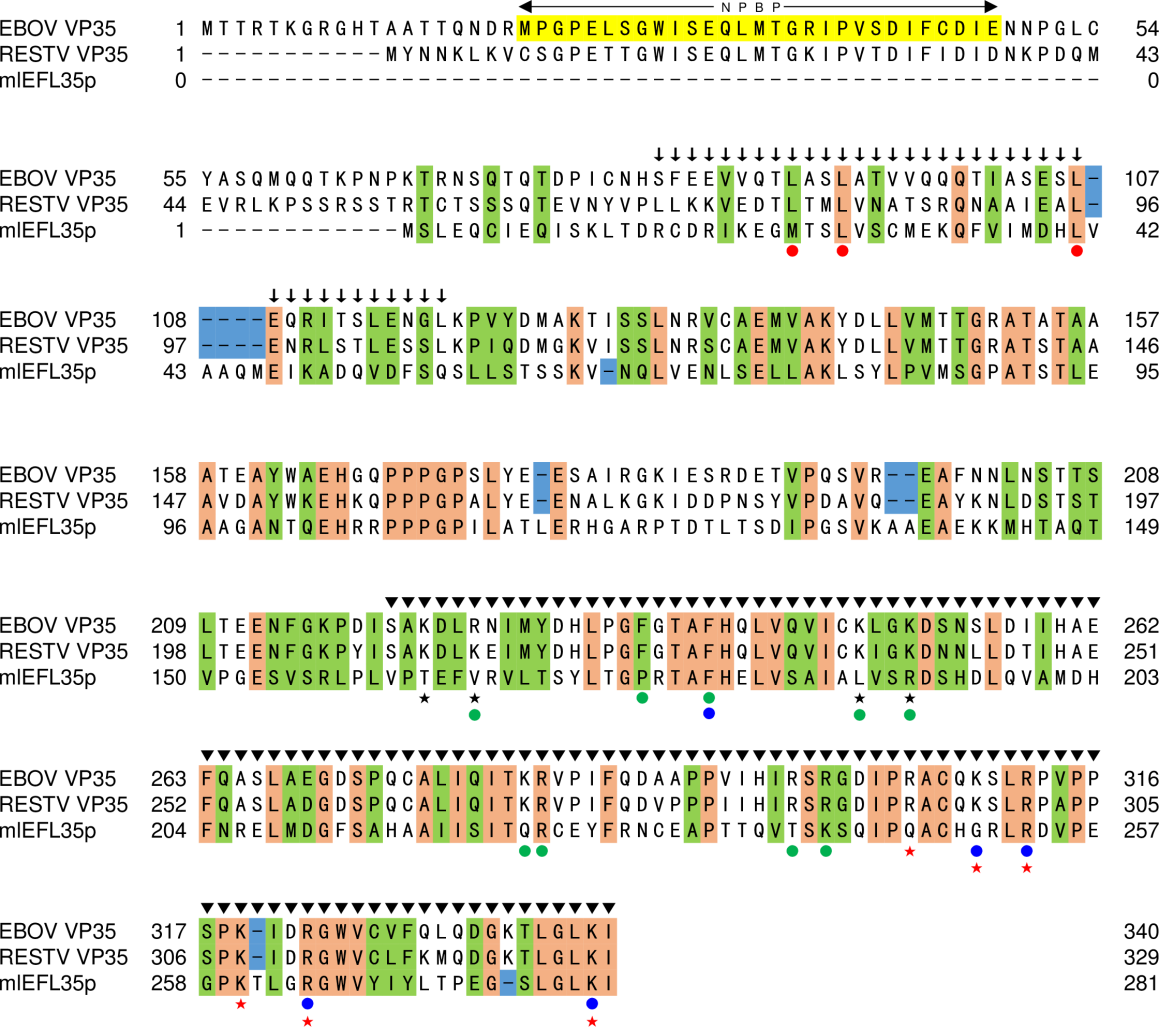
Comparison of primary structures of mlEFL35p and ebolavirus VP35s. The same amino acid residues that are found in VP35s and mlEFL35p are highlighted in orange. Residues highlighted in green represent amino acids that are grouped together in the same classes, based on their physical/chemical properties. The blue rectangles show sequence gaps found between mlEFL35 and VP35s. The NP binding domain consisting of the residues 20–48 (EBOV numbering), termed NPBP, is highlighted in yellow. Amino acid residues indicated by red dots have been identified to be important for VP35 homo-oligomerization as well as viral replication and transcription [35]. Amino acid residues indicated by blue dots and green dots have been shown to be critical for the dsRNA binding and polymerase cofactor activities, respectively [31, 36]. The VP35 homo-oligomerization domain and IFN inhibitory domain are indicated by arrows and arrowheads, respectively. Asterisks indicate cysteine residues in VP35s. Black and red stars indicate the amino acids that form the FBP and the CBP regions on 3-dimensional structure of the VP35 IID, respectively.

We first confirmed that mlEFL35p showed sequence similarities to ebolavirus VP35s with low expectation values with the highest score given by RESTV VP35 (Table 1). We then compared amino acid residues involved in three subdomains mapped on the EBOV and RESTV VP35 sequences [31, 33–36]: the N-terminal domain containing the NP binding peptide (NPBP) at amino acid positions 20–48, the middle oligomerization domain at amino acid positions 82–118 which is required for VP35 homo-oligomerization, and the C-terminal domain, which is called the IFN inhibitory domain (IID), at amino acid positions 220–340 (Fig 1). We found that mlEFL35p completely lacked NPBP in the N-terminal domain. Basic amino acid residues at positions 222, 225, 248, and 251 (EBOV numbering) consisting of the first basic patch (FBP) region which is important for the polymerase cofactor activity [31] were not conserved. Furthermore, of the nine amino acids (i.e., positions 225, 235, 239, 248, 251, 282, 283, 298, and 300) which have been shown to play a critical role in the polymerase cofactor activity [31, 36], only two residues at position 239 and 283 (EBOV numbering) were conserved. Cysteine residues are frequently conserved among evolutionary related proteins that have similar structures [37]. Interestingly, despite the fact that BLASTP hit the Ebola virus VP35 (Table 1), four of the five cysteine residues at positions 135, 247, 275, and 326 (EBOV numbering), were not conserved. On the other hand, leucine residues at positions 93 and 107 (EBOV numbering), both of which are important for VP35 homo-oligomerization [35], were conserved among these proteins. Four (i.e., positions 312, 319, 322, and 339) of the six amino acid residues forming the dsRNA binding site of the IID (e.g., the central basic patch (CBP)) were conserved. These findings suggested that mlEFL35p might lack the ability to interact with NP and might limit the ability to work as a polymerase cofactor, but have a similar biological function to VP35 as an IFN antagonist.

**Table 1:** BLAST search data of mlEFL35.

Program	Query	Database	Subject (Accession Number)	Score	Query cover	E-value^†^	Identity
NCBI BLASTX	mlEFL35	Non-redundant protein sequences	RESTV VP35 (ACT22800)	125	93%	3e^-30^	32%
NCBI BLASTX	mlEFL35	Non-redundant protein sequences	TAFV VP35 (YP_003815424)	104	92%	3e^-22^	30%
NCBI BLASTX	mlEFL35	Non-redundant protein sequences	SUDV VP35 (ACR33188)	102	93%	7e^-22^	31%
NCBI BLASTX	mlEFL35	Non-redundant protein sequences	BDBV VP35 (AGL73451)	102	91%	1e^-21^	33%
NCBI BLASTX	mlEFL35	Non-redundant protein sequences	EBOV VP35 (AKC36417)	102	79%	1e^-21^	33%
NCBI BLASTP	mlEFL35p	PDB	EBOV VP35 (3FKE_A)	84.3	40%	4e^-20^	38%

^**†**^E-value: Expectation-value

### mlEFL35p and VP35s have homo- and hetero-oligomerization potential

**Fig 2:**
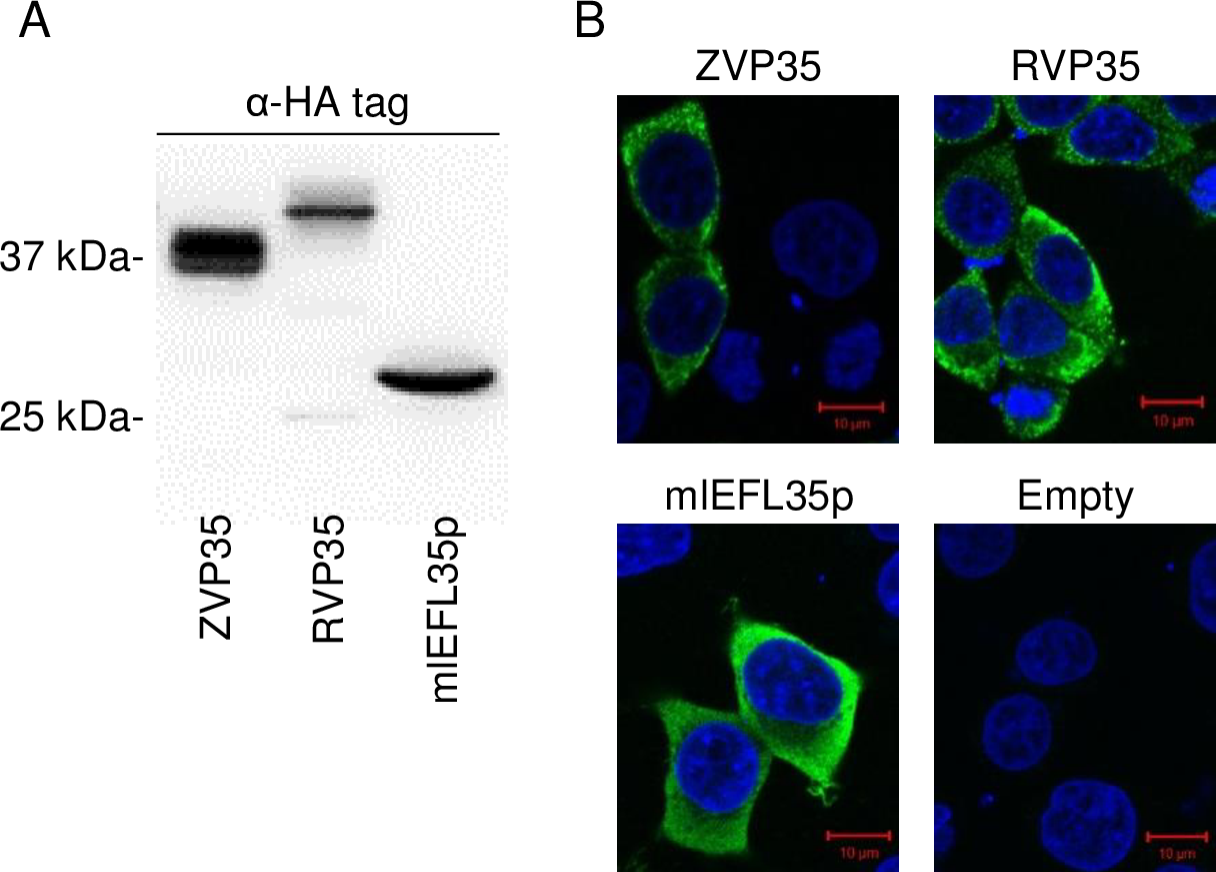
Expression of the mlEFL35p and VP35s in HEK 293T cells. **(A)** Expression of each protein was confirmed by western blotting. HA-tagged mlEFL35p (HA-mlEFL35p), HA-tagged EBOV VP35 (HA-ZVP35) and HA-tagged RESTV VP35 (HA-RVP35) were detected as 30, 37, and 40 kDa proteins, respectively. **(B)** Distribution of each protein is visualized by an immunofluorescence assay with anti-HA antibodies. Cells were counterstained with DAPI.

**Fig 3:**
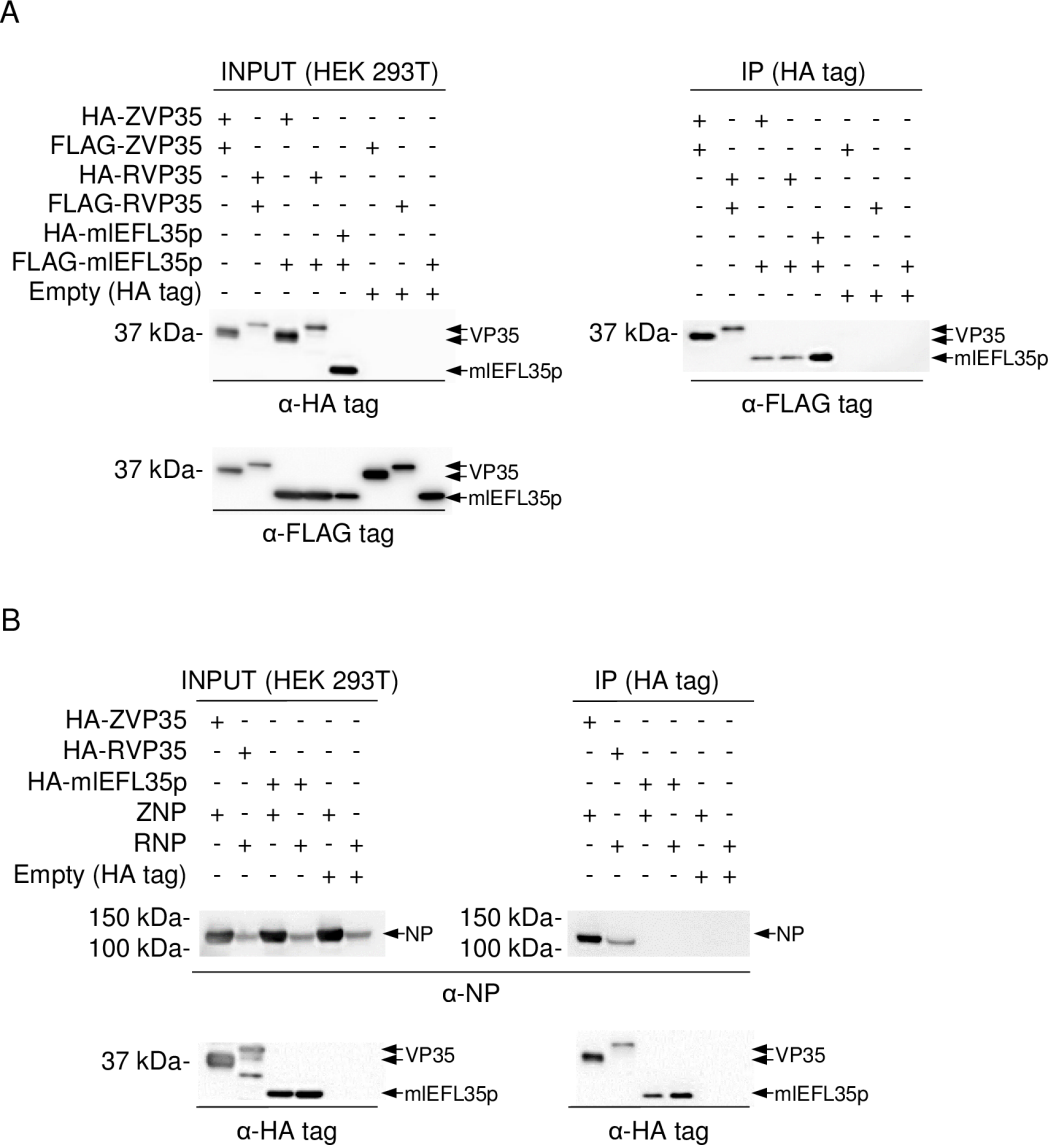
Immunoprecipitation assay of mlEFL35p, VP35s and NP. **(A)** To examine whether mlEFL35p interacted with mlEFL35p itself, EBOV and RESTV VP35s, FLAG-tagged mlEFL35p, EBOV and RESTV VP35s (FLAG-ZVP35 and FLAG-RVP35, respectively) expressed in HEK 293T cells were immunoprecipitated (IP) with HA-tagged mlEFL35p and VP35s of EBOV and RESTV (HA-ZVP35 and HA-RVP35, respectively). Precipitated proteins were detected by western blotting with anti-FLAG tag antibodies. **(B)** EBOV NP (ZNP) and RESTV NP (RNP) were expressed in HEK 293T cells and immunoprecipitated with HA-tagged VP35 of EBOV and RESTV (HA-ZVP35 and HA-RVP35) or HA-tagged mlEFL35p. HA-tagged proteins were detected by western blotting with an anti HA-tag antibody. ZNP and RNP were similarly detected with rabbit antisera specific to NPs.

We transfected human embryonic kidney (HEK) 293T cells with the plasmids expressing mlEFL35p and VP35s of EBOV and RESTV and investigated the expression of these proteins by western blotting and immunofluorescence assays (Fig 2A and 2B). A 30 kDa protein, corresponding to the expected molecular weight of mlEFL35p, was detected by western blotting. In transfected cells, mlEFL35p was detected in the cytoplasm like VP35s and their intracellular localization appeared to be similar. Next, HEK 293T cells were cotransfected with the expression plasmids for HA- or FLAG-tagged mlEFL35, VP35s and/or NP and solubilized proteins were immunoprecipitated with each HA-tagged protein. Consistent with previous reports [31, 33], we found that FLAG-tagged VP35s were coimmunoprecipitated with HA-tagged VP35s irrespective of the ebolavirus species, confirming their homo-oligomerization potential (Fig 3A). Interestingly, FLAG-tagged mlEFL35p was coimmunoprecipitated with not only HA-tagged mlEFL35p but also the HA-tagged VP35s of EBOV and RESTV (Fig 3A). We further analyzed the interactions of mlEFL35p with ebolavirus NPs and found that EBOV and RESTV NPs were coimmunoprecipitated with the HA-tagged VP35s of the respective viruses but not with HA-tagged mlEFL35p (Fig 3B). Taken together, these results indicated that mlEFL35p, like VP35s, had the ability of homo-oligomerization, which might be required for fundamental functions of VP35. However, consistent with its primary structure, mlEFL35p lacked the ability to interact with NP.

### mlEFL35p functions as an antagonist that inhibits the RIG-I-mediated signaling pathway

**Fig 4:**
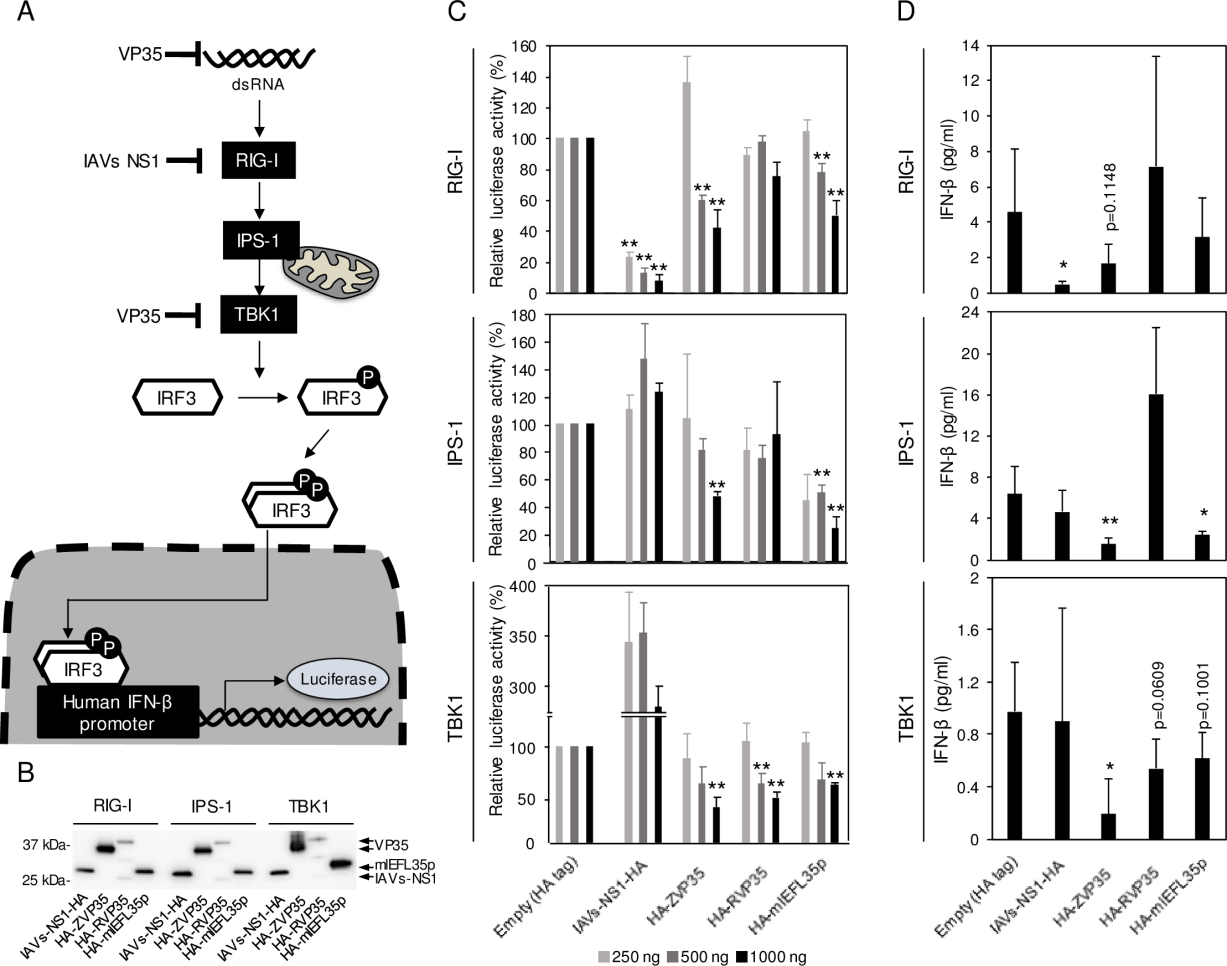
Inhibition of the RIG-I-mediated signaling pathway by mlEFL35 and VP35s. **(A)** The RIG-I-mediated signaling pathway is shown. The human IFN-β promoter is activated through RIG-I, IPS-1 and TBK1. The IFN-β promotor activity was measured by luciferase reporter assays. **(B)** HEK 293 cells were transfected with each plasmid expressing HA-tagged influenza A virus NS1 (IAVs-NS1-HA), EBOV VP35 (HA-ZVP35), RESTV VP35 (HA-RVP35) or mlEFL35p (HA-mlEFL35p) and the plasmids for the reporter gene expression along with the RIG-I CARD domain vector, IPS-1 or TBK1 expression vector. NS1 and VP35 are known as IFN antagonists. Western blotting was performed to examine the expression of NS1, VP35s, and mlEFL35p. Each HA-tagged protein (IAVs-NS1-HA, HA-ZVP35, HA-RVP35, and HA-mlEFL35p) was detected with an anti-HA-tag antibody. (**C**) Transfected cells were solubilized and luciferase assays were performed. Relative luciferase activities were calculated by setting the values given by the cells transfected with a control empty plasmid expressing the HA tag alone. Significantly lower values compared to control cells (Empty) are indicated by asterisks (*p < 0.05, **p < 0.01). (**D**) Concentrations of IFN-β in the supernatants of cells transfected with the indicated plasmids (1000 ng) were measured by ELISA. Means and standard deviations of five independent experiments are shown. Significantly lower values compared to control cells (Empty) are indicated by asterisks (*p < 0.05, **p < 0.01).

The human IFN-β promoter is known to be activated through RIG-I, IPS-1, and TBK1 (Fig 4A) [27]. Using a reporter assay with these activators, the anti-IFN activity of mlEFL35p was evaluated by comparing it to influenza A virus NS1 (IAV NS1), EBOV VP35 and RESTV VP35, all of which are known to act as IFN antagonists (Fig 4B and 4C) [25, 38]. When the human IFN-β promoter activation was induced by RIG-I, mlEFL35p suppressed the IFN-β promoter activity as efficiently as EBOV VP35, but not as well as NS1. IPS-1-triggered IFN-β promoter activation was also inhibited by mlEFL35p at a similar extent to EBOV VP35. Ebolavirus VP35s and mlEFL35p showed similar effects on TBK1-induced IFN-β promoter activity. We then quantified IFN-β in supernatants of transfected cells (Fig 4B and 4D). Consistent with the results of the reporter assay, the concentrations of IFN-β released into the culture supernatant were also decreased in the presence of mlEFL35p. Although a statistically significant difference was observed only between IPS-1-triggered and control cells in the multiple comparison analysis, the expression of mlEFL35p reduced the production of IFN-β significantly or nearly significantly (IPS-1 or TBK1, respectively) when comparisons were made individually between mlEFL35a-expressing cells and negative control cells (Empty). It was noted that the difference in the suppression efficiency between mlEFL35p and VP35s was correlated with that seen in the reporter assay. These results suggested that mlEFL35p could have a potential function as an IFN antagonist.

### mlEFL35p plays a limited role in EBOV genome transcription/replication

**Fig 5:**
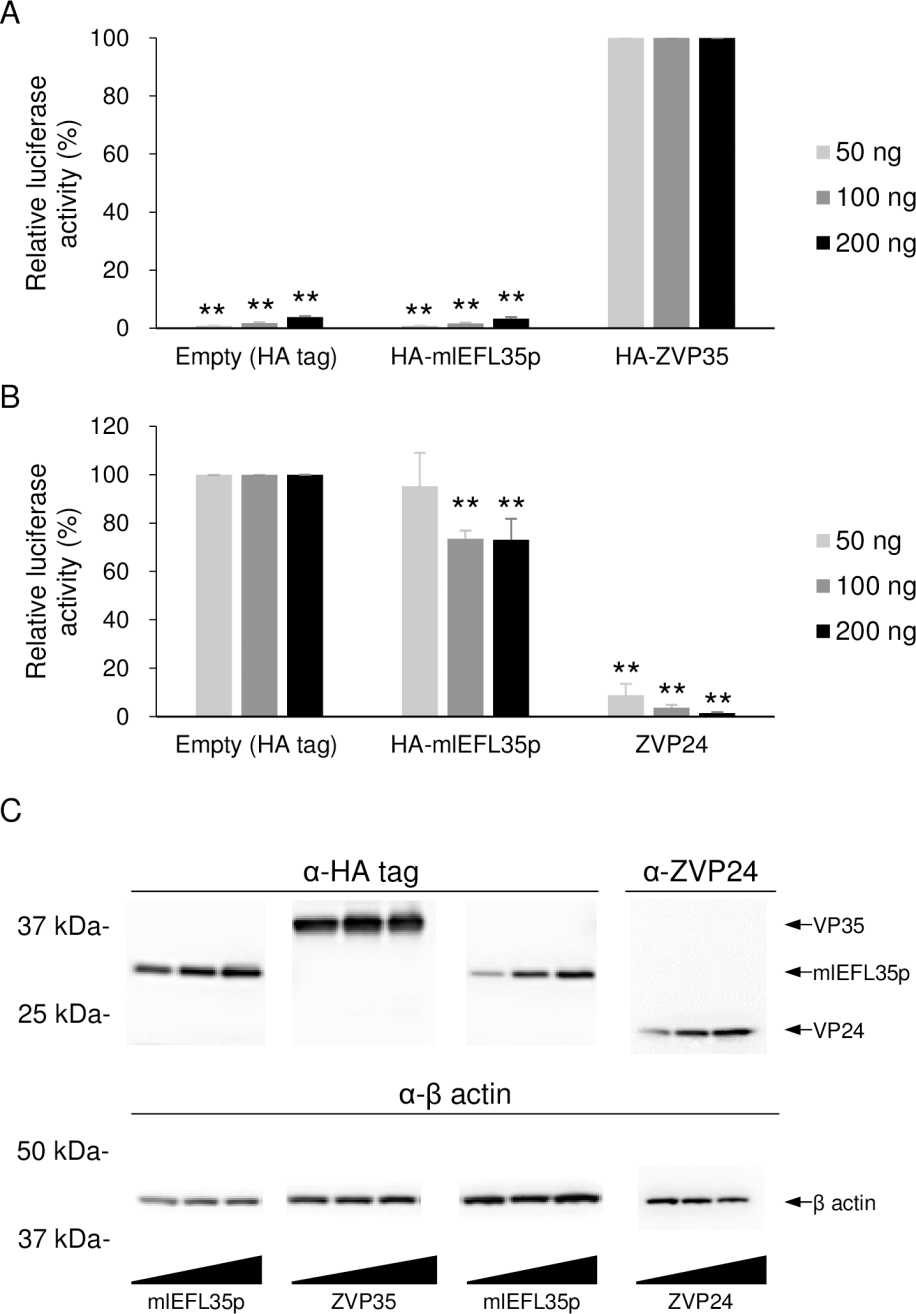
Luciferase expression from the Ebola virus minigenome with mlEFL35p. **(A)** HEK 293T cells were transfected with the indicated amounts of plasmids for the expression of the HA tag alone, HA-tagged mlEFL35p (HA-mlEFL35p), or EBOV VP35 (HA-ZVP35) along with plasmids for the expression of NP, VP30, L, the T7 polymerase and p3E5E-luc. Relative luciferase activities were determined by setting the values of control cells transfected with the HA-ZVP35-expressing plasmid to 100%. Means and standard deviations of three independent experiments are shown. Significant differences from control cells (HA ZVP35) are indicated by asterisks (*p < 0.05). Between the empty control and mlEFL35p, there was no significant difference. **(B)** HEK 293T cells were transfected with the indicated amounts of plasmids for the expression of the HA tag alone, HA-tagged mlEFL35p (HA-mlEFL35p), or EBOV VP24 (ZVP24) along with plasmids for the expression of NP, VP35, VP30, L, the T7 polymerase and p3E5E-luc. ZVP24 was used as a positive control. Means and standard deviations of three independent experiments are shown. Significantly lower values compared to control cells (Empty) are indicated by asterisks (**p < 0.01). **(C)** Expression of each protein was detected by western blotting. HA-tagged proteins (HA-ZVP35 and HA-mlEFL35p) were detected with an anti-HA tag antibody. ZVP24 were detected with a VP24-specific mouse antiserum produced with the synthetic peptide corresponding to amino acid positions 3–15 (KATGRYNLISPKK) of EBOV VP24. β actin were detected with an anti-β actin antibody.

We used the EBOV minigenome system [29] to analyze effects of the mlEFL35p expression on EBOV genome transcription/replication (Fig 5). We first confirmed that EBOV VP35 was required for luciferase expression in this system and then found that the expression of mlEFL35p, in place of EBOV VP35, induced only background levels of luciferase activity given by the empty plasmid (Fig 5A). We further examined the dominant negative effects by overexpression of mlEFL35p (Fig 5B). We found that the expression of EBOV VP24 caused a significant decrease in luciferase activity as shown previously [39]. By contrast, the expression of mlEFL35p only slightly reduced the luciferase activity. Expression levels of the HA-tagged proteins in the transfected cell lysates were analyzed by western blotting (Fig 5C). These results suggested that mlEFL35p might not function as a polymerase cofactor or dominant negative inhibitor in this human cell line.

## Discussion

In this study, we determined the mlEFL35-encoding ORF sequence in the genome of the little brown bat, and biologically analyzed the potential functions of the putative protein, mlEFL35p. Comparison of the amino acid sequences between mlEFL35p and VP35s revealed that the primary structure of mlEFL35p showed high similarity to ebolavirus VP35s. We found that mlEFL35p lacked the NPBP in the N-terminal domain, whereas several amino acid residues important for VP35 homo-oligomerization and the IFN antagonist function were conserved between mlEFL35p and VP35s. Accordingly, we demonstrated that mlEFL35p had the potential to act as an IFN antagonist but not a polymerase cofactor.

As expected from the primary structure (i.e., conserved leucine residues at positions 93 and 107, 4 conserved residues in the CBP), mlEFL35p was coimmunoprecipitated with homologous (mlEFL35p) and heterologous (VP35) molecules, suggesting its homo- and hetero-oligomerization potential. It has been shown that homo-oligomerization of EBOV VP35 is important for its IFN antagonist activity [35]. Our data may also suggest a link between homo-oligomerization of mlEFL35p and its function as an IFN antagonist. In addition, the ability of mlEFL35p to interact with both EBOV and RESTV VP35s strongly suggested that mlEFL35p and VP35s have structural similarity and share some functions. Interestingly, mlEFL35p inhibited the RIG-I-mediated IFN-β production more efficiently than RESTV VP35 and its inhibitory potential was indeed similar to that of EBOV VP35. Although the expression level of RVP35 seemed to be lower than those of ZVP35 and mlEFL35p, since TBK1-triggered IFN-β promoter activation was inhibited as efficiently as ZVP35, it is not highly likely that the low expression of RVP35 was a major cause of less inhibitory potential. However, there is no difference between EBOV and RESTV VP35s in the amino acid residues that are critical for VP35 homo-oligomerization and the dsRNA binding site, both of which are required for the IFN antagonist activity of VP35s [35, 40]. Leung et al. [41] reported that RESTV VP35 contained an additional helical structure in its IFN inhibitory domain and proposed that the helical structure might increase the stability and decrease the flexibility of the RESTV VP35 molecule. Such a structural difference might influence the IFN antagonist activity. On the other hand, Guito et al. [42] demonstrated that both EBOV and RESTV VP35s were similarly potent IFN antagonist proteins. Since differences in experimental conditions (e.g., use of a human codon-optimized ORF of VP35 and different procedures for IFN induction) might cause different effects, further studies are required to clarify whether anti-IFN potential is involved in the differential pathogenicity of EBOV and RESTV.

It has been demonstrated that VP35 interacts with IRF kinases such as TBK1 and inhibitor of κB kinase epsilon (IKKε) and the physical interaction between IKKε and either IPS-1, IRF-3, or IRF-7 was impaired by EBOV VP35 overexpression [43]. As shown in Fig 4, our reporter assay showed that TBK1-induced human IFN-β promoter activity was most significantly inhibited by mlEFL35p, suggesting that mlEFL35p also targets these IRF kinases. EBOV VP35 binds not only dsRNA but also PKR activator (PACT), both of which are recognized by RIG-I. The ability of VP35 to block the PACT activation requires the CBP structure [44]. Comparison of primary structure between mlEFL35p and ebolavirus VP35s (Fig 1) indicates that majority of amino acids forming the CBP structure are conserved in mlEFL35p. This suggests that mlEFL35p may also inhibit the PACT activation like EBOV P35.

It was noted that four of the five cysteine residues in VP35s were not conserved in mlEFL35p. Cysteine residues often form a disulphide bond that plays an important role in protein folding and stability. Thus, cysteine residues important for protein structures are generally conserved among related proteins [37]. Crystal structure analysis has shown that the cysteine residues at positions 247 and 275 of EBOV VP35 do not form a disulfide bond [45]. Although the structural impacts of the other cysteine residues of VP35 remain unknown, our data suggest that cysteine residues of mlEFL35p and VP35s are not important for the fundamental function as an IFN antagonist.

As suggested by the comparison of the amino acid sequences between mlEFL35p and VP35s (e.g., lack of the NPBP region), NP was not coimmunoprecipitated with mlEFL35p. The NP-VP35 interaction has been shown to be essential for viral transcription/replication [29]. Accordingly, our EBOV minigenome reporter assay demonstrated that mlEFL35p could not be substituted for EBOV VP35 in the viral transcription/replication cycle. It was also speculated that overexpression of mlEFL35p might show dominant negative effects by forming hetero-oligomers between mlEFL35p and VP35 molecules. However, mlEFL35p only slightly interfered with the VP35 function. These results suggested that mlEFL35p might not have critical functions involved in viral transcription/replication. Further studies with chimeric proteins between mlEFL35p and VP35 might provide more detailed information on specific regions required for the VP35 function as a polymerase cofactor.

It has been proposed that endogenous retroviruses in animal genomes provide some beneficial effects to host animals and might play important roles in their coevolution [46–48]. It has also been demonstrated that non-retroviral elements, EBLNs, are involved in antiviral effects and regulation of neighboring gene expression [11–13]. However, in this study, we demonstrated that mlEFL35p potentially acted as an IFN antagonist like EBOV VP35, suggesting a suppressive effect on host immunity. While mlEFL35 was found in an insectivorous bat (*Myotis lucifugus*), there is no information on susceptibility of this bat species to ebolaviruses. In addition, cell lines of this bat species are also unavailable. Interestingly, however, some species of fruit bats are suspected to be natural hosts [49–52]. It should also be noted that EBOV is able to replicate and lead to seroconversion without any symptoms in some insectivorous bat species [53]. Overall, previous studies have indicated that many species of bats are susceptible to ebolaviruses. To better understand the potential roles of EFLs in ebolavirus infection, it would be of interest to screen various bat cell lines for the presence of genomic EFLs and to analyze their biological effects by knockdown/knockout experiments using cell lines naturally expressing EFLs. It is reported that transcription of human EBLN-1 is responsible for regulating gene transcription [12–14]. Thus, it is also important to focus on the function of not only EFL-derived proteins but also noncoding RNA transcripts. Once cell lines naturally expressing EFLs are in hand, loss-of-function experiments are needed for further understanding of the biological significance of EFLs in the ebolavirus ecology.

## Materials and methods

### Bioinformatics

The nucleotide sequences of mlEFL35 shown in NCBI (Accession numbers: JN847697 and JN847701) were partial ORFs. To get its full-length ORF, we obtained the genomic sequence between the 5’-target site duplication (TSD) and 3’-TSD from the Ensemble database (http://www.ensembl.org/index.html) since the full-length mlEFL35 ORF was reported to be located between these TSDs [9, 10]. The obtained sequence (1944 nucleotides) was analyzed using the NCBI BLASTX program in the non-redundant protein sequences database and found to contain the mlEFL35 full-length ORF (S1 Text) encoding a putative mlEFL35-derived protein (mlEFL35p) that showed high sequence similarities to ebolavirus VP35s with low expectation values (Table 1). Next, we used the NCBI BLASTP program in the Protein Data Bank to confirm that the amino acid sequence of mlEFL35p matched with the structure of EBOV VP35 (Table 1). Amino acid sequences were aligned using Clustal W [54], and then amino acids having similar physical-chemical properties were grouped.

### Cell culture and construction of plasmids

HEK 293 cells (ATCC^®^ CRL-1573^™^) and 293T cells (ATCC^®^ CRL-3216^™^) were grown in Dulbecco’s modified Eagle’s medium with 10% fetal calf serum (FCS). Cells were incubated in a humidified 5% CO_2_ incubator at 37°C. The cDNA encoding the mlEFL35 ORF was synthesized and cloned into a pUCFa vector (FASMAC). The cDNA encoding mlEFL35p fused to an HA or FLAG tag at the N terminus was cloned into the mammalian expression vector pCAGGS [55] using an In-Fusion cloning kit (BD Clontech). In a similar way, the expression plasmids for N-terminally HA- or FLAG-tagged VP35 and NP of an EBOV isolate, Mayinga (species *Zaire ebolavirus*) or a RESTV isolate, Pennsylvania (species *Reston ebolavirus*) were constructed. The ORF of the NS1 protein of influenza A virus (strain PR8) with a splice acceptor site mutation [56] was C-terminally fused with an HA tag and cloned into pCAGGS. The EBOV minigenome plasmid containing the firefly luciferase gene, p3E5E-luc [39], was also synthesized and cloned into a pUCFa vector (FASMAC). The NP, VP35, VP30, VP24, and L genes of EBOV (Mayinga) were similarly cloned into pCAGGS. Expression vectors used to provide human IPS-1 have been described previously [57]. The human TBK1 gene was also cloned into pCAGGS.

### Western blotting for the detection of expressed proteins

Each cell lysate was mixed with 2 × sample buffer (Bio Rad) and incubated at 65°C for 15 min. Expressed proteins were separated in sodium dodecyl sulfate (SDS)-polyacrylamide gels (SuperSep Ace 5–20%, Wako) and transferred to polyvinylidene fluoride (PVDF) membranes (Merck). PBS containing 3% (wt/vol) skim milk (Becton Dickinson) and PBS containing 0.05% (vol/vol) Tween 20 (PBST) were used as blocking and wash buffers, respectively. PVDF membranes were incubated with an anti-HA monoclonal antibody (abcam, ab1424, 1:5000), anti-β actin monoclonal antibody (abcam, ab6276, 1:5000), or VP24-specific mouse antiserum for 60 min, washed with PBST, and then incubated with horseradish peroxidase-conjugated goat anti-mouse IgG (Jackson ImmunoResearch, 115-035-062, 1:10000) for 60 min. After washing with PBST, the bound antibodies were visualized with Immobilon Western (Millipore).

### Indirect immunofluorescence assay

HEK 293T cells were seeded on 8-well chamber slides (Watson Co., Ltd) precoated with poly-L-lysine (Cultrex). One day after seeding, the cells were transfected with the plasmids encoding mlEFL35p using TransIT LT1 reagent (Mirus) according to the manufacturer’s instructions. At 24 hours post-transfection, the cells were fixed in 4% (wt/vol) paraformaldehyde for 15 min, and permeabilized by incubation for 5 min in PBS containing 0.4% (vol/vol) Triton X-100. The following procedures were performed at room temperature. The cells were incubated with PBS containing 1% (wt/vol) BSA followed by incubation with the anti-HA antibody (abcam, ab1424, 1:500) for 60 min. After washing with PBST, the cells were incubated with Alexa Fluor 488-conjugated goat anti-mouse IgG (Molecular Probes, A11001) for 30 min in the dark. Nuclei were stained using 1 μg/ml 4′,6-diamidino-2-phenylindole, dihydrochloride (DAPI) (Molecular Probes) for 10 min in the dark. Images were acquired with a 63 × oil objective lens on a Zeiss LSM700 inverted microscope and ZEN 2009 software (Carl Zeiss).

### Immunoprecipitation assay

HEK 293T cells were transfected with the plasmids encoding HA- and/or FLAG-tagged VP35 and mlEFL35p using TransIT LT1 reagent (Mirus) according to the manufacturer’s instructions. For the NP expression, pCAGGS plasmids encoding untagged NPs were used. Two days after transfection, the cells were lysed with cold lysate buffer (50 mM Tris-HCl pH 8.0, 150 mM NaCl, 2 mM EDTA, 10% glycerol and 0.05% NP-40) containing EDTA-free protease inhibitor (Roche). To facilitate disruption of the cells, cell suspensions were frozen at -20°C. Samples were centrifuged at 10000 × g at 4°C for 10 min. Supernatants were mixed with EZview Red Anti-HA Affinity Gel beads (Sigma) and incubated at 4°C overnight with gentle rocking. After washing the beads with the lysis buffer, HA-peptides (Sigma, 100 μg /ml) were mixed with them and incubated at 4°C for 15 min with gentle rocking to elute the HA-tagged protein. The beads were centrifuged at 500 × g at 4°C for 1 min and the supernatant was mixed with 2 × sample buffer (Bio Rad) and incubated at 65°C for 15 min. Precipitated proteins were separated in SDS-polyacrylamide gels (SuperSep Ace 5–20%, Wako) and transferred to PVDF membranes (Merck). HA- or FLAG-tagged mlEFL35p and VP35s were detected with the anti-HA tag antibody (abcam, ab1424, 1:5000) or anti-FLAG tag antibody (Sigma, F1804, 1:5000). NPs were detected with a mixture of rabbit antiserum to EBOV NP (FS0169, 1:2000) and RESTV NP (FS0170, 1:2000) [58]. The bound antibodies were visualized with Immobilon Western (Millipore).

### Reporter assay for human IFN-β promoter activity

HEK 293 cells (1 × 10^5^) on 12-well plates were transfected with 250, 500, or 1000 ng of each plasmid expressing C-terminally HA-tagged influenza A virus NS1 (IAVs-NS1-HA), N-terminally HA-tagged EBOV VP35 (HA-ZVP35), RESTV VP35 (HA-RVP35) or mlEFL35p (HA-mlEFL35p) and the plasmids for the human IFN-β promoter-driven firefly luciferase reporter gene (pIFNβ-luc, 250 ng, a kind gift from Sonja Best, NIH/NIAID), the Renilla luciferase-based pRL-TK vector (50 ng, Promega), along with the RIG-I caspase activation and recruitment domain (CARD) domain vector (50 ng/well, a kind gift of Sonja Best), IPS-1 expression vector (50 ng/well), or TBK1 expression vector (100 ng/well) using FuGENE HD transfection reagent (Promega) according to the manufacturer's recommendations. Twenty-four hours after transfection, cell culture supernatants were collected and centrifuged at 300 × g, and then the supernatants were stored at -80°C until use. These cell supernatants were subjected to Enzyme-linked immunosorbent assay (ELISA) to measure the concentrations of human IFN-β. The cells were harvested and lysed in Passive Lysis Buffer (Promega), and then luciferase assays were performed using the Dual-Luciferase Reporter Assay System (Promega) according to the manufacturer's directions. These cell lysates were subjected to SDS- polyacrylamide gels, followed by western blotting, to examine the expression of each protein. Firefly lucifearse values were normalized to Renilla luciferase values. Normalized values were then compared to negative control (no induction) to obtain fold induction values. The results are presented as percent induction in comparison to the positive control (with only the HA-tag expression vector), the value for which was set to 100%.

### Human IFN-β ELISA

The quantitation of IFN-β was examined with a VeriKine-HS Human Interferon Beta Serum ELISA Kit (PBL Assay Science). All procedures were performed according to protocol A of the manufacturer’s instructions. The optical density at 450 nm (OD450) was measured using SoftMax^®^ Pro 6.2.1 software (Molecular Devices). The standard curve was obtained by plotting the OD450, and then the concentrations of IFN-β (pg/ml) in the samples were calculated.

### Minigenome reporter assay

HEK 293T cells (5 × 10^4^) on 24-well plates were transfected with 50, 100, or 200 ng of plasmids encoding the HA tag alone, HA-ZVP35, or HA-mlEFL35p, along with expression plasmids for the production of EBOV NP (50 ng), VP30 (30 ng), L (400 ng), p3E5E-luc (100 ng), and the T7 polymerase (100 ng) using the TransIT LT1 reagent (Mirus). In another experiment, HEK 293T cells (5 × 10^4^) on 24-well plates were transfected with 50, 100, or 200 ng of plasmids encoding the HA tag alone, HA-mlEFL35p, or VP24, along with expression plasmids for the production of EBOV NP (50 ng), VP35 (50 ng), VP30 (30 ng), L (400 ng), p3E5E-luc (100 ng), and the T7 polymerase (100 ng) using the TransIT LT1 reagent (Mirus). At 36 hours after transfection, the cells were lysed with Passive Lysis Buffer (Promega), and the luciferase activity was measured using the Bright-Glo luciferase assay system (Promega) according to the manufacturer’s instructions. These cell lysates were also subjected to SDS- polyacrylamide gels, followed by western blot analysis to examine the expression of each protein. The firefly luciferase activity was compared to the negative control (without EBOV L expression vector) values to obtain fold luciferase activity values and relative luciferase activities were determined by setting the values of control cells to 100%.

### Statistical analysis

All analyses were performed with R (version 3.2.3) [59]. One-way analysis of variance (ANOVA) was performed, followed by a post hoc paired Student's t-test with Bonferroni adjustment for multiple comparisons.

## Supporting information

S1 Text Full-length mlEFL35 ORF and mlEFL35p. (DOCX)
