# Supplementary material for: Putative endogenous filovirus VP35-like protein potentially functions as an IFN antagonist but not a polymerase cofactor
Source: PLoS One. 2017 Oct 17;12(10):e0186450. doi: 10.1371/journal.pone.0186450 (PMC5645129; doi:10.1371/journal.pone.0186450)
Supplement: S1 Text — (DOCX) [file pone.0186450.s001.docx]

**Title**

Putative endogenous filovirus VP35-like protein potentially functions as an IFN antagonist but not a polymerase cofactor

**Short Title**

Functional analyses of endogenous filovirus-like element

**Authors**

Tatsunari Kondoh^1^, Rashid Manzoor^1^, Naganori Nao^1^, Junki Maruyama^1^, Wakako Furuyama^1^, Hiroko Miyamoto^1^, Asako Shigeno^1^, Makoto Kuroda^1^, Keita Matsuno^2,3^, Daisuke Fujikura^4^, Masahiro Kajihara^1^, Reiko Yoshida^1^, Manabu Igarashi^1,3^, and Ayato Takada^1,3,5^*

Affiliation

^1^Division of Global Epidemiology, Research Center for Zoonosis Control, Hokkaido University, Sapporo, Japan

^2^Laboratory of Microbiology, Department of Disease Control, Graduate School of Veterinary Medicine, Hokkaido University, Sapporo, Japan

^3^Global Station for Zoonosis Control, Global Institution for Collaborative Research and Education, Hokkaido University, Sapporo, Japan

^4^Division of Infection and Immunity, Research Center for Zoonosis Control, Hokkaido University, Sapporo, Japan

^5^School of Veterinary Medicine, the University of Zambia, Lusaka, Zambia

*Correspondence and requests for materials should be addressed to Prof. Ayato Takada DVM PhD, Division of Global Epidemiology, Hokkaido University Research Center for Zoonosis Control, Kita-20, Nishi-10, Kita-ku, Sapporo 001-0020, Japan. Phone: +81-11 706-9502, Fax: +81-11 706-7310. Email: [atakada@czc.hokudai.ac.jp](mailto:atakada@czc.hokudai.ac.jp)

**Supporting text**

**>mlEFL35**

ATGTCCCTGGAGCAGTGCATCGAACAGATAAGTAAGCTCACCGATCGCTGTGATAGAATCAAAGAAGGCATGACATCTCTTGTAAGCTGCATGGAAAAGCAGTTTGTTATAATGGATCATCTCGTAGCTGCCCAGATGGAGATAAAAGCAGATCAAGTCGATTTTAGTCAGAGTCTTCTGTCTACGTCTTCTAAGGTTAATCAACTAGTAGAGAATTTGTCTGAGCTTTTAGCCAAGCTTAGTTATTTGCCTGTAATGTCAGGACCTGCAACATCCACTCTCGAGGCAGCTGGAGCAAACACGCAGGAGCATAGAAGGCCTCCCCCAGGGCCCATCCTAGCAACCCTGGAACGACACGGAGCAAGACCCACAGATACTCTCACTTCTGATATTCCAGGGTCCGTGAAGGCTGCTGAGGCCGAGAAGAAAATGCATACTGCACAGACTGTCCCTGGGGAGAGTGTCTCTCGGCTGCCTCTAGTTCCCACCGAATTCGTACGAGTCCTCACAAGTTATCTGACAGGACCGCGCACTGCATTTCATGAATTAGTATCGGCAATCGCTTTGGTGAGCCGAGACTCTCATGATCTACAGGTAGCCATGGACCATTTCAATCGAGAGCTAATGGATGGTTTCTCAGCTCATGCTGCCATAATATCCATCACTCAGAGATGTGAGTATTTTCGGAACTGCGAAGCTCCGACAACGCAGGTAACTTCGAAGAGCCAGATTCCACAAGCATGTCATGGCAGACTTAGGGATGTACCGGAGGGTCCCAAAACCCTAGGACGAGGATGGGTATATATATATCTAACTCCTGAAGGAAGCCTCGGGTTAAAGATTTAA

**>mlEFL35p**

MSLEQCIEQISKLTDRCDRIKEGMTSLVSCMEKQFVIMDHLVAAQMEIKADQVDFSQSLLSTSSKVNQLVENLSELLAKLSYLPVMSGPATSTLEAAGANTQEHRRPPPGPILATLERHGARPTDTLTSDIPGSVKAAEAEKKMHTAQTVPGESVSRLPLVPTEFVRVLTSYLTGPRTAFHELVSAIALVSRDSHDLQVAMDHFNRELMDGFSAHAAIISITQRCEYFRNCEAPTTQVTSKSQIPQACHGRLRDVPEGPKTLGRGWVYIYLTPEGSLGLKI*
